# Tuning network dynamics from criticality to an asynchronous state

**DOI:** 10.1371/journal.pcbi.1008268

**Published:** 2020-09-28

**Authors:** Jingwen Li, Woodrow L. Shew

**Affiliations:** Department of Physics, University of Arkansas, Fayetteville, Arkansas, United States of America; Dartmouth College, UNITED STATES

## Abstract

According to many experimental observations, neurons in cerebral cortex tend to operate in an asynchronous regime, firing independently of each other. In contrast, many other experimental observations reveal cortical population firing dynamics that are relatively coordinated and occasionally synchronous. These discrepant observations have naturally led to competing hypotheses. A commonly hypothesized explanation of asynchronous firing is that excitatory and inhibitory synaptic inputs are precisely correlated, nearly canceling each other, sometimes referred to as ‘balanced’ excitation and inhibition. On the other hand, the ‘criticality’ hypothesis posits an explanation of the more coordinated state that also requires a certain balance of excitatory and inhibitory interactions. Both hypotheses claim the same qualitative mechanism—properly balanced excitation and inhibition. Thus, a natural question arises: how are asynchronous population dynamics and critical dynamics related, how do they differ? Here we propose an answer to this question based on investigation of a simple, network-level computational model. We show that the strength of inhibitory synapses relative to excitatory synapses can be tuned from weak to strong to generate a family of models that spans a continuum from critical dynamics to asynchronous dynamics. Our results demonstrate that the coordinated dynamics of criticality and asynchronous dynamics can be generated by the same neural system if excitatory and inhibitory synapses are tuned appropriately.

## Author summary

What is the dynamical state of cerebral cortex? Are neurons mostly uncorrelated, firing independently of each other? Or are neurons more coordinated, firing in sync with each other? The answers to these questions have fundamental implications for how the cortical neural population encodes and processes information. Here we show that two possible scenarios—criticality and asynchronous dynamics—that are typically considered incompatible can be attained in the same network by maintaining a certain kind of balance, while tuning the strength of inhibition relative to excitation along with the average synaptic strength.

## Introduction

Mounting experimental evidence supports the hypothesis that the cerebral cortex operates in a dynamical regime near criticality [1–9]. In the context of our work here, criticality refers to a boundary in the space of possible dynamical regimes. On one side of the boundary, population activity tends to be orderly with strong correlations among neurons. On the other side, neurons fire more independently of each other resulting in asynchronous population dynamics. At criticality, population dynamics are more diverse, rarely exhibiting synchronization that spans the network, but often showing coordinated firing among groups of neurons at small and intermediate scales [10, 12, 13]. Direct evidence that the cerebral cortex may indeed operate near such a boundary comes from experiments and models in which the balance of excitation (E) and inhibition (I) is disrupted. These studies show that one can push cortical dynamics from a dynamical regime consistent with criticality to a hyperactive synchronous regime by suppressing inhibition (GABA antagonists) or to a low-firing asynchronous state by increasing inhibition (GABA agonists) [14–18]. Also, critical dynamics can be pushed into a low-firing asynchronous regime by suppressing excitation (AMPA and NMDA antagonists) [16–18]. These observations support the hypothesis that the cortex may operate near criticality under normal conditions, but only if the proper balance of E and I is maintained.

However, not all observations of the cortex under ‘normal conditions’ exhibit the diverse multi-scale coordination that is expected near criticality. Indeed, many experimental measurements have revealed relatively asynchronous firing, particularly in vigilant and active behavioral conditions [19–24]. The stark difference between the observations of coordinated critical dynamics and asynchronous dynamics has traditionally fueled a debate about which is a better description of the cortex.

A prominent class of models, often referred to as ‘balanced networks’, offers an explanation of this more asynchronous activity. Beginning with the ‘chaotic balanced state’ hypothesis [25, 26], the idea is that E and I inputs to any given neuron wax and wane together, nearly canceling each other most of the time. During brief moments the E-I cancellation is imperfect and neurons can fire, contributing to an asynchronous and irregular activity. Over last two decades, numerous numerical and theoretical studies have addressed the dynamics and function of balanced networks (a few examples include [27–31]). Experiments supporting this possibility show the balance between excitation and inhibition based on whole cell recordings of E and I inputs [32–35].

Both critical dynamics and asynchronous dynamics have been observed in awake animals and both seem to require balanced E and I. However, the difference in coordination of population activity for criticality versus asynchronous activity is stark. How can we reconcile these two types of neural activity? When should we expect to see the coordination of criticality; when should we expect to see asychronous activity?

Recent computational modeling efforts have begun to tackle these questions. They have shown several different ways that tuning one or a few simple parameters can result in a shift from asynchronous dynamics to critical dynamics or vice versa [24, 36–38]. For example, Priesemann and colleagues showed that tuning the input and an effective branching parameter (*m* in their terminology) can generate a family of models ranging from fully asynchronous to critical [11, 36]. Although very useful, this model was too abstract to identify specific biological mechanisms that might be responsible for changing *m*. Buendia et al. studied networks of excitatory and inhibitory neurons and found that, for sufficiently sparse networks, a new regime emerges near criticality with weak flucutations around a moderate mean firing rate, reminiscent of some asynchronous activity [37]. Dahmen et al. also studied sparse networks of excitatory and inhibitory neurons, highligting how increasing the heterogeneity of synaptic strengths can change the dynamics of a system from traditional criticality (as discussed here) to a different type of critical regime at the “edge of chaos” which manifests with asynchronous dynamics [24]. Most recently, Girardi-Schappo et al. studied a more complex model of probabilistic leaky integrate and fire neurons [38], showing that Brunel’s classic parameter space [39] includes a critical point that is adjacent to the ‘asynchronous regular’ regime and nearby the ‘asynchronous irregular’ regime studied by Brunel and others. (Touboul and Destexhe have pointed out that the ‘synchronous irregular’ state in Brunel’s model is not consistent with criticality [40]). These models suggest a revision of the traditional debate; instead of asking which is a better description of cortical dynamics—criticality or asynchronous dynamics—we should acknowledge that cortex can generate both types of dynamics. Our work here builds on this premise, with the goal of exploring possible models of how a single cortical network might shift between critical dynamics and asynchronous dynamics.

We hypothesize that criticality requires a different kind of E/I balance than that needed to generate asynchronous activity. We address this possibility using a network-level model of probabilistic, binary neurons. By tuning excitatory and inhibitory synaptic strengths (keeping network structure and the input to the network fixed), we find that we can generate a family of models, spanning a continuum from criticality to the asynchronous networks. When synapses are strong and balanced, asynchronous network activity results. When synapses are relatively weak and balanced, criticality results. Our results offer a possible explanation for the variety of experimental observations, suggesting that the cortex could shift its dynamical regime from near criticality to asynchrony and a continuum of intermediate states between these extremes, all while maintaining a certain type of balanced excitation and inhibition.

## Results

We study population dynamics of a recurrent network of *N* = 1000 probabilistic binary neurons, similar to models used in previous studies of criticality [16, 41, 42]. There are 800 excitatory neurons and 200 inhibitory neurons. We consider how several aspects of dynamics depend on changes in excitatory and inhibitory interactions. More specifically, we tune two parameters: the relative strengths of excitatory and inhibitory synapses (the I/E weight ratio) and the average synaptic weight.

**Fig 1:**
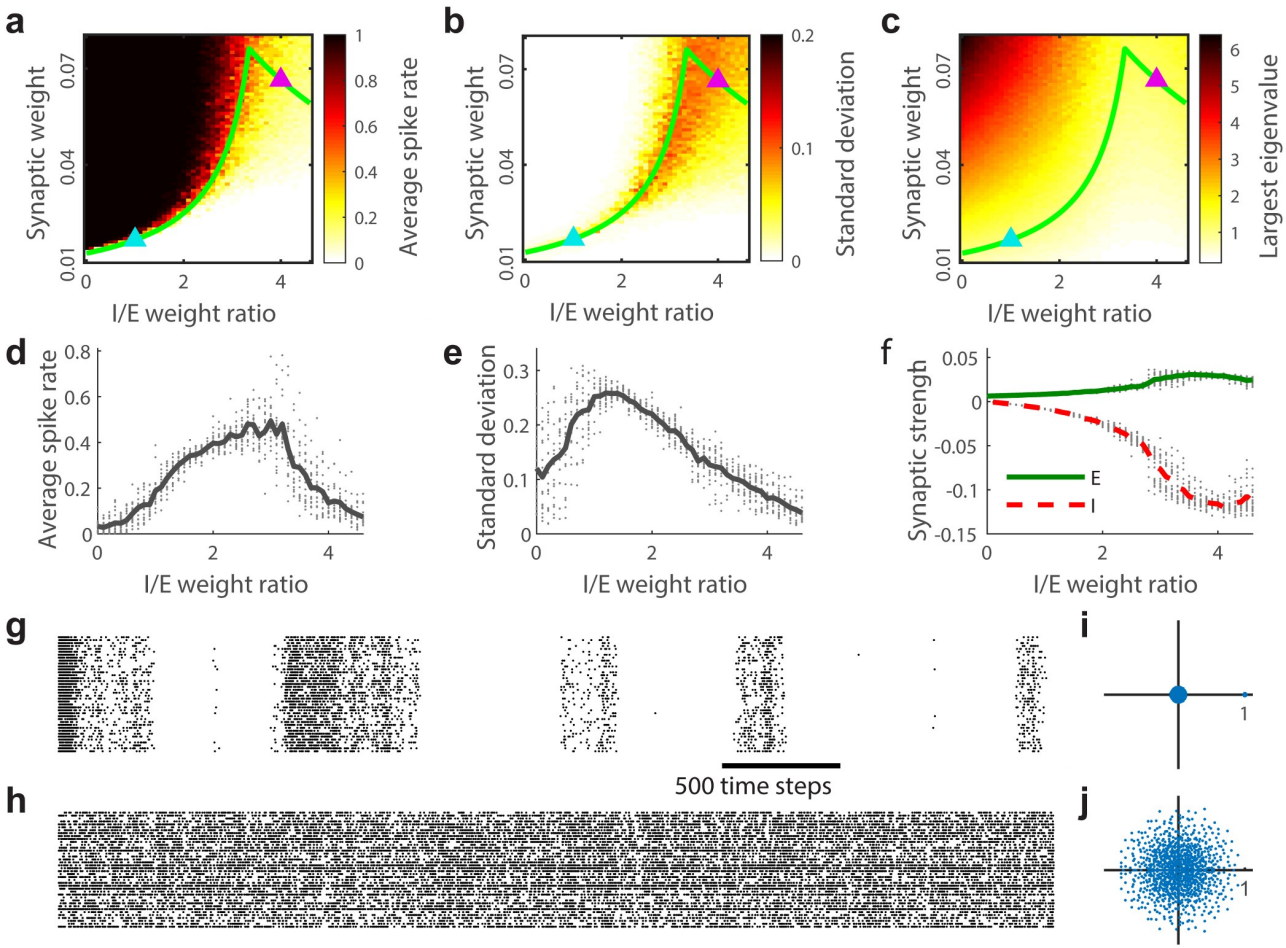
Synchronous and asynchronous dynamical regimes along the λ_*max*_ = 1 boundary. **(a)** Time averaged population spike rate as a function of I/E weight ratio and synaptic weight. The λ_*max*_ = 1 boundary (green line) divides a high firing rate regime (black) from a low firing rate regime (white). **(b)** Standard deviation of the population spike rate across time as a function of I/E weight ratio and synaptic weight. Fluctuations are largest along the λ_*max*_ = 1 boundary. **(c)** The largest eigenvalue λ_*max*_ of the synaptic weight matrix as a function of I/E weight ratio and synaptic weight. λ_*max*_ > 1 in the high firing rate regime and λ_*max*_ < 1 in the low rate regime. **(d)** Shown is the spike rate along the λ_*max*_ = 1 boundary. The spike rate is moderate all along the boundary. **(e)** Shown is the amplitude of spike rate fluctuations (standard deviation across time) along the λ_*max*_ = 1 boundary. **(f)** Excitatory and inhibitory synaptic weights balance each other but become stronger as I/E weight ratio is increased along the λ_*max*_ = 1 boundary. **(g,h)** Example spike rasters for the low and high I/E ratio regimes, corresponding to the blue and pink triangles in panels a-c, respectively. **(i,j)** Eigenvalue spectra for the two examples in panels g and h.

The majority of the two-dimensional parameter space for our model is occupied by either a high firing rate regime with dominant excitation or a low firing rate regime with dominant inhibition (Fig 1a). Both of these imbalanced regimes are poor models for real cortical network dynamics, which have more moderate firing rates. Along the boundary between the high and low firing rate regimes more realistic dynamics occur; neither E nor I dominates and firing rates are moderate (Fig 1d). In this paper we study the dynamics along this boundary line in more detail.

One way to identify the boundary between the high and low firing rate regimes more precisely is to consider the eigenvalue spectrum of the connection matrix. The largest eigenvalue along the boundary is equal to 1 (green line in Fig 1a–1c). The high and low firing rate regimes have the largest eigenvalues greater and less than one, respectively (Fig 1c). One implication of having the largest eigenvalue λ_*max*_ equal to 1 is that activity does not systematically grow nor decay on average as time passes, which is a reasonable requirement for modeling real brain activity. Another way this requirement could be met is with λ_*max*_ somewhat less than 1, but with stronger external input. We emphasize that our model is well known to operate at criticality when parameters are set such that λ_*max*_ = 1 and I/E = 0 (i.e. when there are only excitatory neurons). This is known from many previous studies using a similar model [16, 17, 41]. However, with a few exceptions [42], how our model dynamics deviate from criticality when the I/E ratio is substantially increased is less well understood.

**Fig 2:**
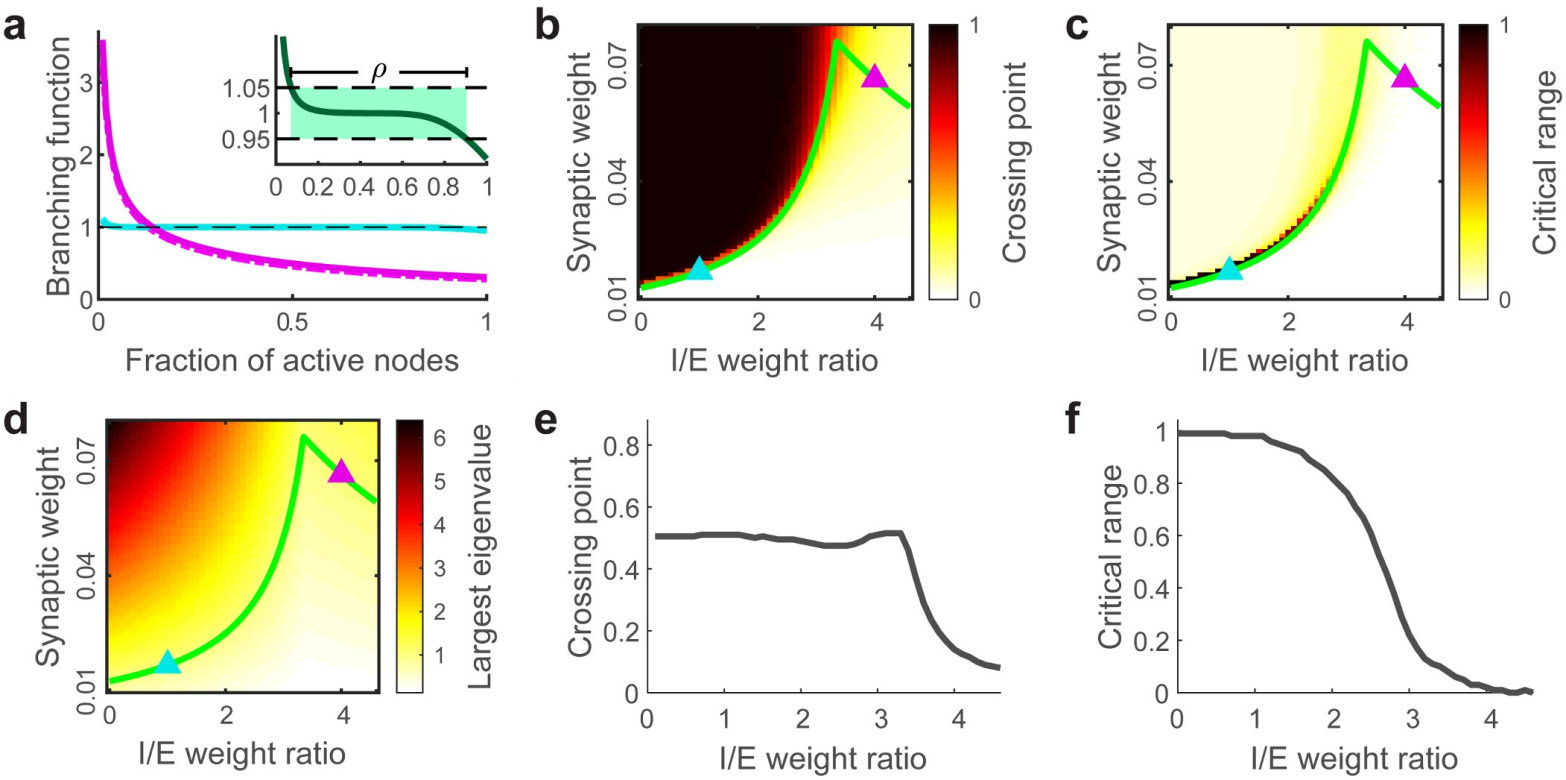
Analysis of branching functions and eigenvalues explain model behavior. **(a)** Branching functions Λ are distinctly different for networks in the synchronous regime (blue) and asynchronous regime (pink). Solid lines are calculated semi-analytically and dash lines are obtained from simulating the model. Inset: The critical range *ρ* is defined as the range of *S* for which Λ(*S*) lies between 1.05 and 0.95. **(b)** Crossing point where Λ = 1 as a function of I/E weight ratio and synaptic weight, which successfully reproduces average spike rate in simulations. **(c)** Critical range as a function of I/E weight ratio and synaptic weight. The large critical range at low I/E with weak synaptic weight is consistent with the high standard deviation of population activity in the synchronous regime. **(d)** The theoretical largest eigenvalue of the synaptic weight matrix as a function of I/E weight ratio and synaptic weight derived in Eqs 1–3. Green line in panels b-d indicates the λ_*max*_ = 1 boundary. **(e)** The value of the crossing point where Λ = 1 along the λ_*max*_ = 1 boundary. **(f)** The value of the critical range along the λ_*max*_ = 1 boundary.

We find that the nature of population activity varies dramatically along the λ_*max*_ = 1 boundary. For weak synapses and smaller I/E ratios the λ_*max*_ = 1 boundary is sharply defined and coordinated fluctuations are large in amplitude (Fig 1a–1h). For stronger synapses and higher I/E ratios, the boundary is less sharply defined, activity is asynchronous, and fluctuations are less prominent(Fig 1a–1h). The eigenvalue spectra of the weak-synapse regime and the strong-synapse regime also differ qualitatively (more detailed analysis of eigenvalue spectra are in Methods). The eigenvalue spectrum in the weak-synapse regime is characterized by a single positive real outlying eigenvalue (Fig 1i). The eigenvalue spectrum in the strong-synapse regime consists of circular cloud in the complex plane without any single dominant outlying eigenvalue (Fig 1j). Between these extremes, at a particular point along the λ_*max*_ = 1 boundary, there is a cross-over between these two types of model characteristics. The cross-over occurs where the I/E weight ratio equals to 4 in the large *N* limit. In our model with *N* = 1000, the cross-over occurs near 3.34 (Fig 2d), which we confirm analytically in Methods (Eq 4). Note however, that this cross-over point does not locate the boundary of critical dynamics; critical dynamics do not emerge unless the I/E weight ratio is smaller, below 1 approximately.

In the remainder of this paper we will examine differences between these two regimes: the synchronous regime at a low I/E weight ratio and weak synaptic strength, and the asynchronous regime at a high I/E weight ratio and strong synaptic strength. We will examine several aspects of dynamics traditionally associated with criticality including branching functions [43] and neuronal avalanche size and duration distributions [14, 43, 44]. We will also examine several aspects of dynamics related to asynchronous neural dynamics including correlation functions and cancellation of E and I inputs [20, 27, 32]. In agreement with previous work, we will show that the weak-synapse λ_*max*_ = 1 boundary corresponds with criticality, while strong synapses and λ_*max*_ = 1 result in asynchronous dynamics.

The ‘branching function’ was developed in previous work to theoretically analyze population activity near criticality [43]. This approach using the branching function is an alternative to using mean field theory, which fails to characterize the dynamical properties when the I/E weight ratio is low [30]. First, we define *S*(*t*) as the fraction of neurons in the network that are firing at time *t*. The branching function Λ(*S*) is defined as the expected value of *S*(*t* + 1)/*S*(*t*) conditioned on the level of activity *S*(*t*). In the weak-synapse, synchronous regime, the branching functions have a wide range near Λ(*S*) = 1 (blue line in Fig 2a), which means the system is able to wander through various population firing rates. This ‘flat’ branching function has been considered as a hallmark of criticality in previous work [43]. In contrast, if the branching function has a substantial slope, crossing Λ(S*) = 1 at a particular value of *S* = S*, then the firing rate is relatively stable with small fluctuations around *S* = S* (e.g. pink line in Fig 2a). Using a semi-analytical method to determine the shape of the branching function (Methods), we are able to obtain good qualitative predictions (Fig 2b and 2e) of the average spike rates that we observe when we run our model (Fig 1a). In the synchronous regime, the population activity often fluctuates greatly around the mean, as expected due to a wide range near Λ(*S*) = 1 in the branching function. We define the ‘critical range’ *ρ* to measure the range of the branching function that stays within a certain distance of 1 (Fig 2a inset). The large critical range in the synchronous regime (Fig 2c and 2f) fits reasonably well with the large standard deviation in simulations (Fig 1b). In conclusion, the weak-synapse regime has large critical range, while the strong-synapse regime has small critical range.

**Fig 3:**
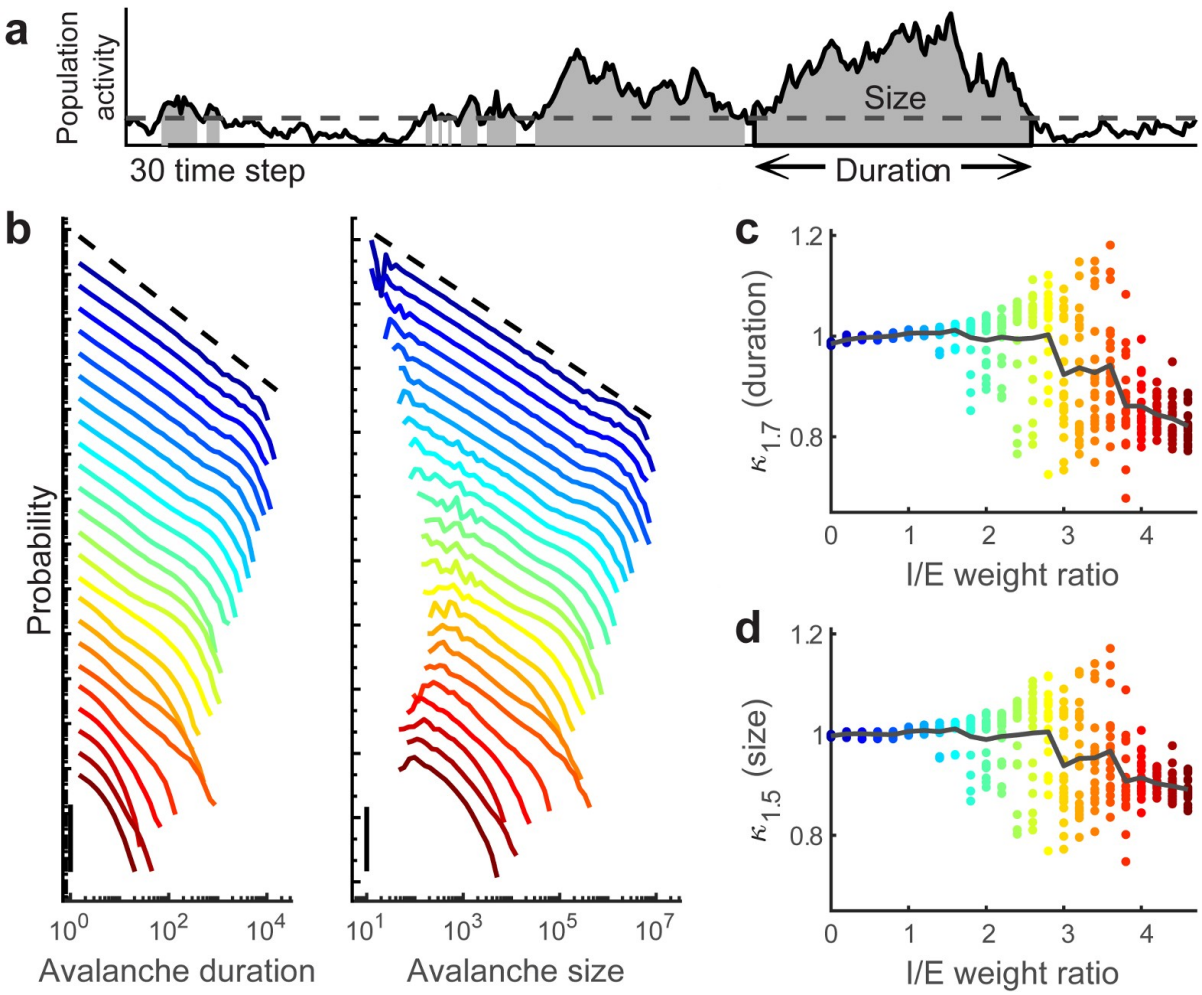
Avalanche distributions indicate deviation from criticality as I/E ratio is increased. **(a)** An avalanche is defined as a time period during which the number of active neurons (solid line) exceeds a threshold (dashed line). Avalanche duration is the number of time steps included in an avalanche, while avalanche size is the number of spikes included in an avalanche. **(b)** The probability distributions of avalanche duration and size for different I/E weight ratios. Color represents different I/E weight ratios as in panels c and d. Vertical axis is logarithmic with the scale bar showing 3 orders of magnitude. Distributions are shifted vertically for comparison. **(c)** Deviation from a power law with exponent −1.7 is measured by *κ*_1.7_ for avalanche duration as a function of I/E weight ratio. **(d)** Deviation from a power law with exponent −1.5 is measured by *κ*_1.5_ for avalanche size as a function of I/E weight ratio. For each I/E, we generate multiple realizations (dots) and average them to obtain the mean (solid line).

As mentioned above, it is well known that our model operates at criticality when λ_*max*_ = 1 and I/E = 0 (i.e. in the lower left part of our parameter space). The branching functions with large critical range for the weak-synapse regime are consistent with this fact. Another expectation near criticality is that distributions of neuronal avalanche sizes and durations should have power-law form [14, 44, 45]. Next we study how these power-law avalanche statistics break down as I/E is increased. Similar to previous work [14, 43, 46], we define an avalanche as a period of time during which the number of active neurons exceed a threshold (Fig 3a). The duration and size of an avalanche are defined as the number of time steps and the total number of spikes that occurred during the avalanche, respectively. Next, we examine avalanche distributions along the λ_*max*_ = 1 boundary (green line in Figs 1 and 2). We find that both avalanche duration and size distributions change dramatically along this boundary (Fig 3b). When the I/E weight ratio is low, the avalanche duration and size distributions are close to power-law distributions, as expected at criticality. As the I/E weight ratio increases, the distributions of avalanche duration and size deviate from power-law distributions, with large avalanches becoming less prominent. Similar to previously developed methods [5, 14, 16, 46], we use *κ*_*ϵ*_ to quantify how much a distribution deviates from a power-law distribution with exponent −*ϵ*. If the distribution is close to the power-law distribution, then *κ*_*ϵ*_ is close to 1, which occurs for both avalanche duration and size distributions when the I/E weight ratio is low. Based on the power law exponent we observe for low I/E, we consider *ϵ* = 1.5 for the size distributions and *ϵ* = 1.7 for the duration distributions. Any deviation in *κ*_*ϵ*_ from 1 means a deviation from the power-law distribution. As the I/E weight ratio grows larger, *κ*_*ϵ*_ starts to deviate from 1, then varies erratically for intermediate I/E before settling near *κ*_*ϵ*_ = 0.8 as I/E approaches 4. Since power-law avalanche size and duration distributions are a necessary condition for criticality, we conclude that our model deviates from criticality as the I/E weight ratio is increased along the λ_*max*_ = 1 boundary.

**Fig 4:**
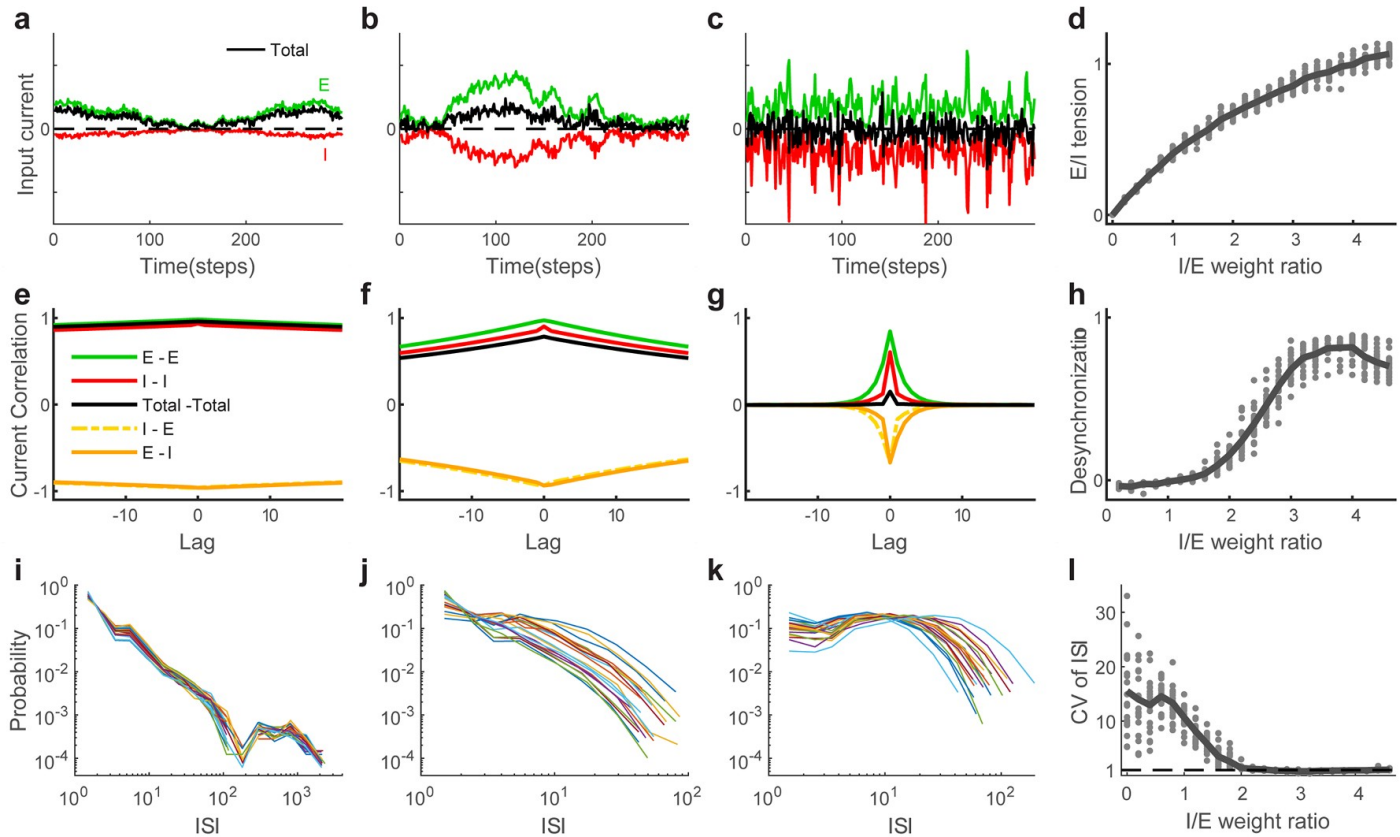
Excitatory inputs cancel inhibitory inputs in asynchronous regime for high I/E. **(a-c)** Synaptic inputs when the I/E weight ratios are 1, 2 and 4, respectively. The excitatory (green) and inhibitory (red) inputs are shown separately from the total synaptic input (black). **(d)** E/I tension as a function of the I/E weight ratio. For each I/E, we generate multiple realizations (dots) and average them to obtain the mean (solid line). **(e-g)** Population-averaged cross correlograms (CCGs) of total synaptic inputs (black), excitatory inputs (green) and inhibitory inputs (red) when the I/E weight ratios are 1, 2 and 4, respectively. **(h)** The level of desychronization *η* as a function of I/E weight ratio. For each I/E, we generate multiple realizations (dots) and average them to obtain the mean (solid line). **(i-k)** Distributions of inter-spike-intervals (ISI) are similar across neurons for the low I/E regime, more varied across neurons for the high I/E regime. **(l)** The coefficient of variation (CV) of ISI as a function of the I/E weight ratio. CV of ISI is high for small I/E and approaches 1 as the I/E weight ratio is increased. For each I/E, we generate multiple realizations (dots) and average them to obtain the mean (solid line).

Considering the decrease in critical range and deviation from power-law avalanche distributions, and previous work with similar models [16, 41, 42], we conclude that our model deviates from criticality as we tune it from the weak-synapse synchronous regime to the strong-synapse asynchronous regime. Next, we study properties predicted by some theories to occur in asynchronous neural activity. We first examine the excitatory and inhibitory inputs to the model cells. For all I/E ratios, E and I inputs are strongly correlated, but as we increase the I/E ratio, the dynamics are tuned from a state in which excitatory input dominates (is not canceled by inhibition) to a state in which inhibitory input cancels the excitatory input more and more exactly (Fig 4a–4c). We define ‘E/I tension’ to measure how tightly the excitatory and inhibitory inputs cancel each other (Methods). Fig 4d shows that the E/I tension gradually increases as the I/E weight ratio increases, and reaches as high as 1 when the I/E weight ratio is 4. This result is consistent with previous work showing that tightly balanced E and I currents canceling each other leads to asynchrony [27].

Another essential property of asynchronous population activity is that neurons should be weakly correlated across neurons and across time. Thus, we next examine the population-averaged input cross-correlogram (CCG) for different I/E weight ratios (Fig 4e–4g). When the I/E weight ratio is low, CCGs of excitatory, inhibitory and total inputs all reveal strong correlations across neurons and over long timescales. As the I/E weight ratio increases, CCGs decrease in amplitude and timescale. When the I/E weight ratio is high, although the excitatory and inhibitory input CCGs remain relatively high at zero delay, the total input CCG is weak due to balance between excitatory and inhibitory inputs. We quantify asynchrony based on decreases in temporal and cross-neuron correlations. For this, we define *η* to be inversely proportional to the area under CCG for total input (normalized as defined in the Methods). As shown in Fig 4h, asynchrony *η* sharply increases when the I/E weight ratio goes beyond 2, and reaches a high value when the I/E weight ratio is over 3, consistent with the turning point in the eigenvalue analysis. Therefore, strong inhibition makes excitatory and inhibitory inputs balanced and leads to asynchronous activity. This is in accordance with the result in previous work that the population-averaged firing correlation is weak when inhibition is strong and fast [20]. Considering together the balance of E and I inputs and the asynchronous firing, we conclude that high I/E with strong synaptic strength in our model is consistent with two prominent expectations for asynchronous network activity.

Finally, we consider the coefficient of variation (CV) of inter-spike-intervals (ISI). For Poisson firing, which is often associated with asynchronous dynamics, the CV of ISI is near 1, while more bursty firing will have CV of ISI greater than 1. We find that in the weak-synapse, synchronous regime, ISI distributions are similar across neurons, while in the strong-synapse, asynchronous regime, ISI distributions are more varied across neurons (Fig 4i–4k). Moreover, as I/E weight ratio is increased, the CV of ISI (averaged across neurons) is high for low I/E ratios, decreasing to near 1 in the asynchronous regime (Fig 4l).

## Discussion

We have shown that the population activity of neural networks can vary dramatically depending how excitation and inhibition are balanced. If weak excitation is balanced by weak inhibition, we found that the dynamics exhibit large fluctuations and rather coordinated activity at criticality. If stronger inhibition balances stronger excitation in a higher “tension” balance, we found that the dynamics are asynchronous and steady. Along with other recent studies [24, 36–38], our findings suggest that the same cortical network could be tuned from criticality to asynchrony, by tuning inhibition and excitation appropriately.

How do our results compare to other recent studies relating critical dynamics to asynchronous dynamics? In the work of Priesemann and colleagues, they found that critical dynamics can shift toward asynchronous dynamics when they reduced the efficacy of activity propagation (their *m* parameter), while increasing the input to the network [11, 36]. They have also shown how homeostatic plasticity mechanisms can act to give rise to a range of dynamical regimes including criticality and asynchronous states, depending on the input to the system [47]. In contrast, in our model, we kept input to the network fixed while strengthening synapses to tune the network from criticality to asynchrony. Since stronger synapses are more effective for propagating activity, we suspect our work points to a different mechanism than that discussed by Priesemann and colleagues. Buendia et al. focused on the ‘low-activity intermediate’ (LAI) regime, which had small fluctuations similar asynchronous regimes [37]. Their LAI regime emerged when they reduced the density of connections to a sufficiently sparse level (less than 0.01). In our model the density of connections was 0.2, which suggests that our model is dealing with a different type of asynchronous regime. Our observed changes in eigenvalue spectra as we tuned our model from criticality to asynchrony are similar to those studied by Dahmen et al. when they compared two types of critical dynamics [24]. As the outlying real eigenvalue comes closer to the bulk of complex eigenvalues, while maintaining a largest eigenvalue near 1, traditional criticality (like that discussed here) is replaced with “edge of chaos” criticality. Like our case, this change in eigenvalues and dynamics was also accompanied by changes in the relative strengths of excitatory and inhibitory synapses in their model. These similarities suggest that the asynchronous regime in our model might correspond to “edge of chaos” criticality, but, considering the differences in their model (firing rate) and ours (probabilistic binary neurons), a more careful study would be required to test this possibility. Finally, we note that Girardi-Schappo et al. found that a network of probabilistic leaky-and-fire neurons can be tuned from criticality to an ‘asynchronous irregular’ regime (as defined by [39]) by strengthening inhibition relative to excitation (increasing their *g* parameter) [38]. Our model, considered together with the work of Girardo-Schappo et al. and Dahmen et al. suggests that there may be a general principle governing our models; perhaps increasing the strength of inhibition relative to excitation, while maintaining balance, will always result in a shift away from criticality, towards asynchronous dynamics. Additional theoretical work will be required to test this possibility.

In our work here, our goal was to start with a model that is well-understood to operate at criticality and then push that model away from criticality to generate asynchronous dynamics. Other studies have approached a similar question starting from models with well-understood asynchronous dynamics and pushing them into regimes with larger population-level fluctuations (but not criticality). For example, Ostojic found that the asynchronous balanced state can become unstable with large population-level fluctuations (termed the ‘heterogeneous asynchronous state’) [30]. They implemented this change by increasing synaptic strengths, starting with λ_*max*_ < 1 and resulting with λ_*max*_ > 1. Other recent studies have extended asynchronous balanced networks to regimes with coherent activities [31, 48]. However, none of these studies approached the critical regime.

One interesting hypothesis that emerges from our work concerns metabolic efficiency. First, we note that maintaining a “strong” synapse depends on metabolically expensive biophysical mechanisms—greater presynaptic vesicle pool, greater density of postsynaptic receptors, etc. Since the high I/E regime and the low I/E regime have similar mean firing rates, it stands to reason that the strong synapses of the high I/E scenario would consume more metabolic resources than the lower I/E scenario. Moreover, the critical dynamics we observed at low I/E are associated with a number of functional benefits [4, 13, 14]. On the other hand, the lower fluctuations found in the high I/E regime may be beneficial for functions that require lower “noise” [4, 25, 27]. When low noise is not required, perhaps the brain could tune itself to the low I/E regime where energy consumption is less. This is consistent with the observation that resting, awake animals tend to exhibit greater fluctuations in population activity compared to alert, active animals. Experimental tests of this idea would be challenging, requiring comparisons of synaptic strengths across behavioral states. We would predict that alert, active states would exhibit stronger synapses (i.e. excitatory and inhibitory postsynaptic potentials that are larger in magnitude) than those found in quiescent, resting states.

Our model, in agreement with other recent models, suggest that a single cortical network can shift between two dramatically different dynamical regimes that have traditionally been viewed as incompatible: criticality and asynchronous dynamics. By bridging the gap between these two points of view, we are optimistic that our results help resolve the debate over what kinds of dynamical regimes can manifest in the cortex.

## Methods

### Network architecture and eigenvalue spectrum

We construct the recurrent network with *N* = 1000 binary neurons. Interactions among neurons are determined by an *N* × *N* connection matrix *J*, with the synapse from neuron *i* to neuron *j* specified in row *j* and column *i*. Each neuron randomly connects to each other neuron with a probability *p* = 0.2 similar to that found in experiments [49, 50] (disconnected neurons have a 0 entry in *J*). A fraction *α* = 0.2 of the neurons are inhibitory with outgoing synapse strengths drawn from a uniform distribution in [0, −*gw*]. The rest of the neurons are excitatory, with outgoing synapse strengths drawn from a uniform distribution in [0, *w*]. Thus, the connectivity matrix *J* is governed by two parameters, *w* and *g*, where excitatory synaptic strength is *w*/2, on average, and inhibitory synaptic strength has the average −*gw*/2.

Based on eigenvalue spectrum analysis [51–53], the connectivity matrix has two real eigenvalues determined by the global strength and balance of excitation and inhibition. One is 0 and another one is a non-trivial eigenvalue

$$\begin{array}{c} \lambda_{b}=\frac{w}{2}Np\left(1-\alpha \right)-\frac{gw}{2}Np\alpha \text{.} \end{array}$$ (1)

The other eigenvalues are related to the substructure of the matrix and scattered within a circular cloud centered at the origin on the complex plane with radius

$$\begin{array}{c} R=\sqrt{N[\left(1-\alpha \right)\sigma_{E}^{2}+\alpha \sigma_{I}^{2}]}\;\text{,} \end{array}$$ (2)

where $\sigma_{E}^{2}=(\frac{p}{3}-\frac{p^{2}}{4})w^{2}$ and $\sigma_{I}^{2}=(\frac{p}{3}-\frac{p^{2}}{4}){\left(gw\right)}^{2}$ are the variance of all the elements in excitatory sub-matrix and inhibitory sub-matrix, respectively. Thus, the largest eigenvalue of the connectivity matrix is

$$\begin{array}{c} \lambda_{max}=\max(\lambda_{b},R)\text{.} \end{array}$$ (3)

Whether λ_*b*_ or *R* is larger depends only on the I/E weight ratio *g*, and is independent of the synaptic strength *w*. Setting λ_*b*_ = *R*, we have

$$\begin{array}{c} g^{*}=\frac{p\left(1-\alpha \right)\alpha +\sqrt{p^{2}{\left(1-\alpha \right)}^{2}\alpha^{2}-[p\alpha^{2}-(\frac{4}{3}-p)\alpha /N][p{\left(1-\alpha \right)}^{2}-(\frac{4}{3}-p)\left(1-\alpha \right)/N]}}{p\alpha^{2}-(\frac{4}{3}-p)\alpha /N}\text{,} \end{array}$$ (4)

which depends on the network size *N*. With the parameters in our model, the switching point is near 3.34. At large limit *N*, we have g* = (1 − *α*)/*α* = 4.

### Dynamics

We apply probabilistic dynamics on the recurrent network. The state of each neuron is binary, either 1 or 0 corresponding to active or quiescent, respectively. At each time step *t*, the probability of neuron *i* being active depends on two independent factors: a probability *p*_*i*_ due to synaptic inputs from other neurons within the network and a probability *p*_ext_ due to external inputs or spontaneous firing.

$$\begin{array}{c} \begin{array}{cc} & p_{i}\left(t\right)=\{\begin{array}{cc} 1 & \;\text{for}\;I_{i}\left(t\right)\geq 1\\ I_{i}\left(t\right) & \;\text{for}\;0\leq I_{i}\left(t\right)<1\\ 0 & \;\text{for}\;I_{i}\left(t\right)<0 \end{array}\text{,}\\ & I_{i}\left(t\right)=\sum_{j=1}^{N}J_{ij}s_{j}\left(t-1\right)\text{,} \end{array} \end{array}$$ (5)

where *s*_*j*_(*t* − 1) is the state of neuron *j* at time step *t* − 1, and *I*_*i*_(*t*) represents the total synaptic input to neuron *i* from other neurons at time step *t*. *p*_ext_ is set as 0.005/*N*, which corresponds to 1 externally-driven spike every 200 time steps over the whole network on average. We chose a low rate of external input, because high rates of external input are known to preclude critical dynamics [3]. In simulation, we first apply *p*_*i*_ on each neuron and then apply *p*_ext_ independently. We run the simulations on the networks in Matlab 2010a.

### Branching function

Branching function Λ(*S*) is defined as the expected value of *S*(*t* + 1)/*S*(*t*) for a given level of activity *S*:

$$\begin{array}{c} \Lambda \left(S\right)=\text{E}[S(t+1)|S(t)=S]/S\text{,} \end{array}$$ (6)

where *S*(*t*) is the fraction of active neurons at the time step *t*. Λ(*S*_0_)>1 means that when the activity *S* = *S*_0_ the firing rate tends to grow, on average, while Λ(*S*_0_)<1 means the firing rate tends to decrease when *S* = *S*_0_. We obtain the branching function numerically by running the model one time step forward many times for each possible value of *S*(1), and averaging the ratio of *S*(2)/*S*(1). We also obtain a prediction for the branching function by combining the definition of branching function and the dynamics in our model.

$$\begin{array}{c} \Lambda \left(S\right)=\frac{1}{S}E[\sigma (\sum_{i=1}^{n_{E}}J_{E_{i}}-\sum_{j=1}^{n_{I}}J_{I_{j}})]\text{,} \end{array}$$ (7)

where *n*_*E*_ and *n*_*I*_ are the number of active presynaptic excitatory and inhibitory neurons, respectively. Here, *σ* is a step-wise linear function with the same form as used the to obtain the firing probability *p*_*i*_ = *σ*(*I*_*i*_(*t*)) above. $J_{E_{i}}$ and $J_{I_{j}}$ represent the *i*th excitatory and *j*th inhibitory synaptic strength. Considering the synaptic strength on average, we have

$$\begin{array}{c} \begin{array}{cc} \Lambda (S) & =\frac{1}{S}E[\sigma (\frac{w}{2}n_{E}-\frac{gw}{2}n_{I})]\\ & =\frac{1}{S}\sum_{n_{E}=1}^{N}\sum_{n_{I}=1}^{N}P\left(n_{E}\right)P\left(n_{I}\right)\sigma (\frac{w}{2}n_{E}-\frac{gw}{2}n_{I})\text{.} \end{array} \end{array}$$ (8)

We obtain a good prediction for Λ(*S*) by assuming *n*_*E*_ and *n*_*I*_ are Poisson variables with means *μ*_*E*_ = *Np*(1 − *α*)*S* and *μ*_*I*_ = *NpαS*, respectively.

Critical range is defined as *ρ* = *S*_2_ − *S*_1_, which measures how long the branching function stays near 1. *S*_1_ and *S*_2_ are the fractions of active nodes when Λ(*S*_1_) = 1.05 and Λ(*S*_2_) = 0.95, as shown in Fig 2a insert.

### Avalanche distribution

We define the threshold for avalanches at the level *S*^†^ when the branching function Λ(*S*^†^) = 1.01, as done in previous work [43]. We use *κ*_*ϵ*_ to measure how much the avalanche duration and size distributions deviate from power-law distributions with exponent −*ϵ* [17]. *κ*_*ϵ*_ is defined as

$$\begin{array}{c} \kappa_{\epsilon }=1+\frac{1}{10}\sum_{i=1}^{10}F_{\epsilon }^{\text{NA}}\left(\beta_{i}\right)-F\left(\beta_{i}\right)\text{,} \end{array}$$ (9)

where $F_{\epsilon }^{\text{NA}}$ is the cumulative distribution function (CDF) of the reference power-law distribution with exponent −*ϵ*, and *F* is the CDF of the avalanche duration or size. From the definition, *κ*_*ϵ*_ is close to 1 if the measured distribution matches the reference power-law distribution well. *κ*_*ϵ*_ > 1 means the measured distribution tends to have more large avalanches than the reference distribution. *κ*_*ϵ*_ < 1 means the measured distribution tends to have fewer large avalanches than the reference distribution. We take *β*_*i*_ as a representative sample of 10 logarithmically spaced points along the measured distribution. We use *ϵ* = 1.5 for avalanche size and *ϵ* = 1.7 for avalanche duration, which are the best-fitted exponents with *g* = 0 and λ_*max*_ = 1. 1.5 has been widely observed as the exponent for avalanche sizes at criticality [44, 54, 55]. It has also been theoretical derived in similar models with no inhibition, operating at criticality [56].

### Synaptic input

The total synaptic input *I*_*i*_(*t*) can be separately examined in two parts: excitatory synaptic input $I_{i}^{\text{E}}\left(t\right)$ where only positive connections *J*_*ij*_ > 0 count, and inhibitory synaptic input $I_{i}^{\text{I}}\left(t\right)$ where only negative connections *J*_*ij*_ < 0 count. We define the E/I tension *T* to measure how tightly the $I_{i}^{\text{E}}\left(t\right)$ and $I_{i}^{\text{I}}\left(t\right)$ are balanced. The E/I tension of neuron *i* is defined as

$$\begin{array}{c} T_{i}=1-\frac{{\langle I}_{i}\left(t\right)\rangle }{\langle I_{i}^{\text{E}}\left(t\right)\rangle +\langle |I_{i}^{\text{I}}\left(t\right)\left|\right\rangle } \end{array}$$ (10)

where 〈⋅〉 indicates time average. Then, the E/I tension *T* of the recurrent network is the average of *T*_*i*_ over all neurons.

### Input cross-correlogram (CCG)

We plot the input CCGs by calculating the population-averaged cross correlation function of synaptic inputs with time lags from −20 to 20 time steps. To measure the the degree of asynchrony of the total synaptic input from the synchronized excitatory and inhibitory synaptic inputs, we define *η* as

$$\begin{array}{c} \eta =1-\frac{A_{\text{Total}}}{(A_{\text{EE}}+A_{\text{II}})/2}\text{,} \end{array}$$ (11)

where *A*_Total_ is the area under total synaptic input CCG, and *A*_EE_ and *A*_II_ are the area under excitatory and inhibitory synaptic input CCGs, respectively.
